# Editorial: Editors’ showcase 2021: Insights in cell growth and division

**DOI:** 10.3389/fcell.2023.1208767

**Published:** 2023-05-09

**Authors:** Philipp Kaldis

**Affiliations:** ^1^ Department of Clinical Sciences, Clinical Research Centre (CRC), Lund University, Malmö, Sweden; ^2^ Lund University Diabetes Centre, Clinical Research Centre (CRC), Lund University, Malmö, Sweden

**Keywords:** cell and developmental biology, cell cycle, fertility, protein degradation, DNA damage

The Research Topic “Insights in Cell Growth and Division” is a collection of articles published in the Cell Growth and Division section of Frontiers in Cell and Developmental Biology (https://www.frontiersin.org/journals/cell-and-developmental-biology). Since this journal views the topic of cell and developmental biology quite broadly, it only makes sense that the articles cover a lot of ground. This includes ovarian primordial-to primary follicle transition, DNA damage checkpoint, neural stem cell cycle regulators, asymmetric cell division, the mammary gland, protein degradation, functions of centrosomes, tumor suppressors, centrioles, and genome and chromosome stability (for more detail see below). While, several articles deal with cell cycle in the broadest sense, others focus on development.

For example, Thompson et at. discuss the functions of SKP1, a component of the SCF (SKP1, Cullin 1, F-box protein) complex in genome and chromosome stability and how it relates to cancer. Following a similar path, Guerber et al. present an overview of the Ubiquitin Binding Protein 2-Like (UBAP2L), which plays several roles in cancer including promoting cell proliferation, growth, EMT, migration, invasion, metastasis, vascularization, and survival.

Centrioles form centrosomes and are important components of cells, which is the topic of two reviews. Langlois-Lemay and D’Amours discuss the functions of centrosomes beyond their traditional role of microtubule organizing centers touching on cell cycle progression, DNA damage, sensory reception, and cell homeostasis. Avidor-Reiss et al. investigate the functions of centrioles in male fertility in a variety of mammals. Continuing with the theme of fertility, Ataei-Nazari et al. studied the functions of CDK6, a well-known cell cycle regulator, in oocyte development and define a new role for CDK6 in the primordial-to-primary transition. Lee reviews the mammary gland in regard to shared pathways in embryonic mammary gland cells and breast cancer.

There are three articles related to cell cycle regulation including the DNA damage checkpoint, asymmetric cell division, and neural stem cell cycle regulators. Yam et al. describe a DNA damage checkpoint at the G2/M transition with regards how it engages and how it is switched off. Mistakes in this checkpoint leads to adaptation where the cells continue to divide despite the damage. Li et al. discuss asymmetric cell division which plays an important role in stem cells. They focus on cancer stem cells and whether asymmetric cell division contributes to tumor heterogeneity and cancer progression. Caron et al. review neural stem cell cycle regulators in zebrafish. Zebrafish is an excellent model system to study neurogenesis and neuroregeneration which identified niche-specific cell cycle behavior and novel cell cycle regulators.

Finally, Bourouh and Marignani cover the Liver kinase B1 (LKB1), an important regulator of metabolism and cancer. They focus on lung cancer and the aberrant metabolic pathways connected to LKB1 loss.

Overall, this Research Topic contains well-written articles by experts in their field that will be of great interest to a broad audience. At the same time, it shall remind us that cancer is primarily a signaling disease as was aptly described by Yaffe (2019).
